# Multiple myeloma identified within the same site of the mandible with medication-related osteonecrosis of jaw: An unusual case report

**DOI:** 10.1097/MD.0000000000034260

**Published:** 2023-07-21

**Authors:** Xiao-Hong Wu, Shi-Wei Chen

**Affiliations:** a Fujian Key Laboratory of Oral Diseases and Fujian Provincial Engineering Research Center of Oral Biomaterial and Stomatological Key Lab of Fujian College and University, School and Hospital of Stomatology, Fujian Medical University, Fuzhou, China.

**Keywords:** bisphosphonates, case report, mandibular, multiple myeloma, osteonecrosis

## Abstract

Multiple myeloma (MM) is a malignant disease characterized by abnormal proliferation of plasma cells, which usually occurs in middle-aged and elderly male patients. Bisphosphonates (BP) are commonly used for the treatment of MM bone disease. Long-time use of BP may cause medication-related osteonecrosis of the jaw (MRONJ). MRONJ occurs in jaw exclusively, and Multiple myeloma can also invade the jaw. The 2 diseases have similar clinical manifestations and imaging findings. This report present a case of MM identified in surgical specimen at the site that had been previously pathologically diagnosed as MRONJ in a patient with MM.

**Patient concerns:**

A 57-years-old male patient visited our clinic on October 16, 2020 because of gingival swelling and pain in the right mandible for 1 month after extraction of the lower right premolar. The patient had a long-time illness history of multiple myeloma, and received intravenous zoledronic acid treatment.

**Diagnoses:**

Based on the clinical characteristics, imaging, and pathological findings of sequestrum formation and high inflammatory cell infiltration, the patient was diagnosed with MRONJ. After 1 year, a mandibular osteotomy was performed and pathological analysis showed the presence of necrotic bone and a large number of abnormal plasma cell infiltration, suggesting the presence of MM in the mandible.

**Interventions:**

The patient was treated with a series of conservative treatments including antibiotic treatment, saline irrigation and laser irradiation, as well as superficial sequestration was. One year later, a mandibular osteotomy was performed.

**Outcomes:**

For the patient, the symptoms of gingival swelling, pain and discharge disappeared after surgery.

**Lessons:**

These findings suggested MRONJ and MM could occur simultaneously at same site, so patients with MM presenting with symptoms of MRONJ should be screened for concurrent or disease relapse of multiple myeloma to prevent misdiagnosis or inadequate management. Meanwhile, this also suggests long-term inflammatory may lead to invasion of multiple myeloma.

## 1. Introduction

Multiple myeloma (MM) is a malignant tumor characterized by the accumulation of malignant plasma cells in the bone marrow and the secretion of large amounts of nonfunctional immunoglobulins. MM mostly occurs in middle-aged and elderly men aged 50 to 60 years.^[1]^ Among patients with MM, approximately 79% have osteolytic bone disease.^[2]^ MM occurs in jaw with an incidence of 14% to 30%.^[3,4]^ The oral presentation can be the first sign or the manifestation of MM development. Bisphosphonates are recommend to prevent skeletal-related events in patients with MM, of which zoledronic acid is first choice. However, long-term administration of bisphosphonates, particularly intravenous administration, has been associated with 4% incidence of medication-related osteonecrosis of the jaw (MRONJ).^[5]^ The rate of MRONJ in patients with MM was about 8.5%, much higher than that of patients with breast cancer or prostate cancer,^[6]^ and tooth extraction was an important risk factor.

MRONJ occurs in jaw exclusively, and MM can also invade the jaw. The 2 diseases have similar clinical manifestations and imaging findings. There are several reports about MM disguised as MRONJ,^[7–11]^ in which the cases were initially diagnosed as MRONJ clinically but were later confirmed to be MM through pathological analysis, while simultaneous finding of MRONJ and MM in same site has been rarely reported. In this report, the patient was previously clinically and pathologically diagnosed as MRONJ, but 1 year later, MM was found pathologically in the surgical specimen that involved with MRONJ.

## 2. Case report

A 57-years-old male patient was referred to our department in October 2020, because of recurrent gingival pustules on the lower right mandibular after tooth extraction. The patient had been diagnosed with multiple myeloma in 2014 and was put on VAD (vincristine, doxorubicin, dexamethasone) treatment, without family history of MM. In 2018, anterior and posterior debridement plus internal fixation of the thoracic spine (T7) tumor was performed, followed by long term administration of thalidomide and dexamethasone and zoledronic acid (4 mg every 4 weeks) for 24 months. Regular review showed that his condition was relatively stable.

In mid-February 2020, the patient experienced bilateral low back pain, and was again put on zoledronic acid from June 2020 for a total of 5 instillations. During this period, the symptoms of multiple myeloma were well controlled. In September 2020, the residual root of tooth #45 was extracted in a dental clinic for it was painful. However, 1 month after the extraction, the patient complaint of painful gingival swelling.

## 3. Examination

**Figure 1.**
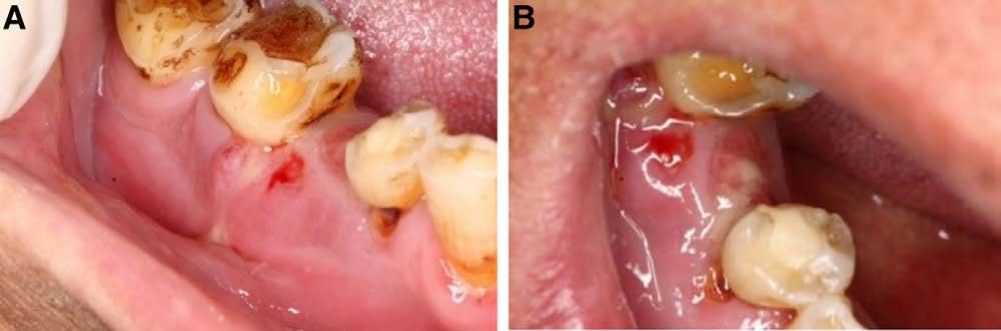
(A and B) Gingiva pus of the edentulous area of the lower right second premolar.

**Figure 2.**
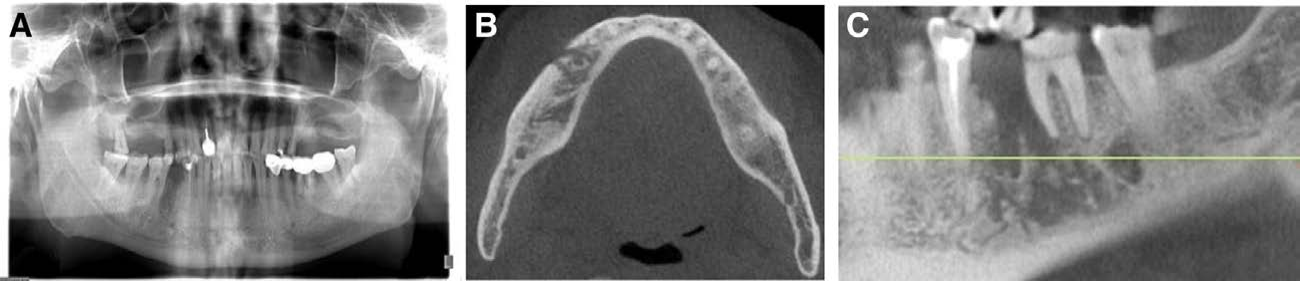
(A-C) Panoramic film and CBCT showing sequestrum formation in the non-healing lower right extraction socket with disrupted cortical plates. CBCT = Conebeam computer tomography.

**Figure 3.**
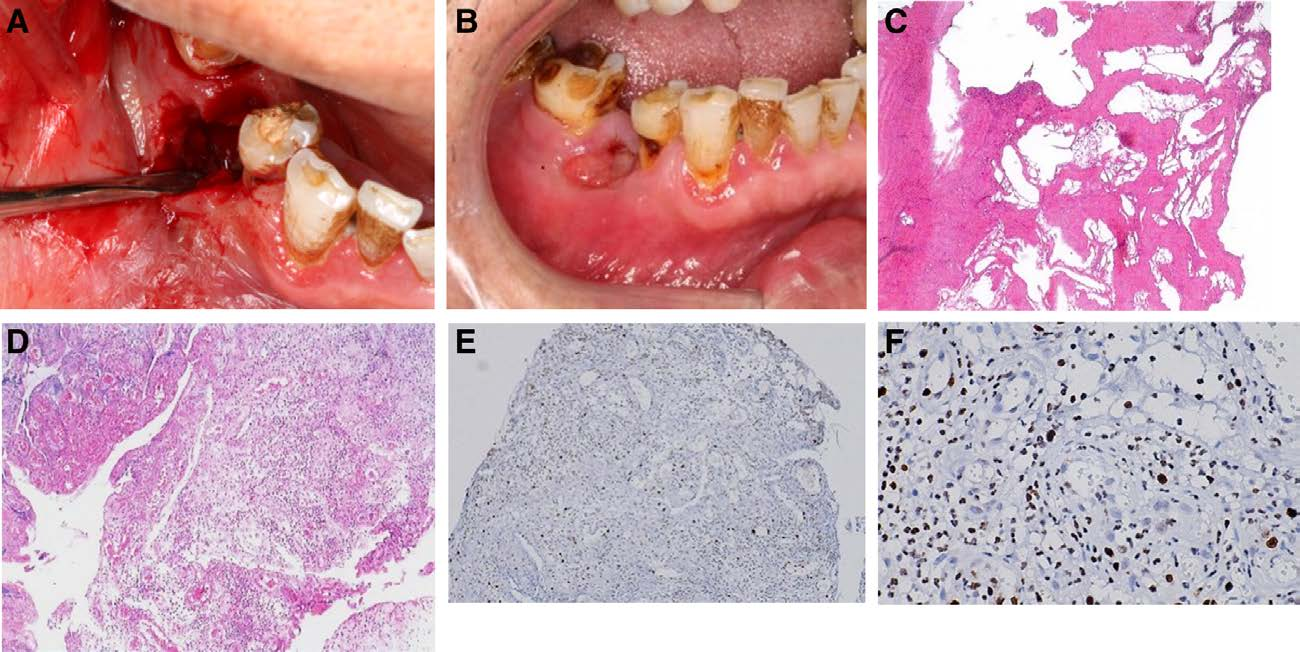
(A) Debridement under flap surgery. (B) 1 month after surgery, the gums healed well. (C) necrotic bone was observed in which the osteocyte had disappeared. (D) High infiltration of inflammatory cells in gingival tissue. (E and F) A few plasma cell in the background with no obvious light chain imbalance, and no tumor cells were found in biopsy.

When the patient first visited our clinic on October 16, 2020, we found a fistula under the extraction socket of the lower right premolar and the buccal apex of lower first molar with pus and black fluid over flowing from the compressed gingiva (Fig. 1A and B). Panoramic film and Dental Conebeam computer tomography (CBCT) showed the formation of granular sequestrum in the right mandible, and the edge of bone destruction was not clear (Fig. 2). Based on the medical history, clinical examination, and imaging data, the patient was diagnosed with stage II MRONJ according to the guidelines outlined in the American Association of Oral and Maxillofacial Surgeons position paper published 2014. The patient then received a series of conservative treatments, such as saline irrigation and topical mouthwash, use of amoxicillin (500 mg tid) and metronidazole (500 mg tid). Er:YAG laser irradiation through gingiva fistula was also conducted. After 2 weeks of follow-up, the patient’s fistula was free of pus, the redness and swelling had subsided. However, the pus reappeared after 1 month. In December 2020, considering the poor efficacy of conservative treatment, with the patient’s consent, superficial sequestrum debridement was performed to remove the sequestrum (Fig. 3A and B), which was taken together with some granulation tissue for pathological analysis. The pathological results showed sequestrum formation and high infiltration of inflammatory cells (Fig. 3C–F), which further supported MRONJ diagnosis.

**Figure 4.**
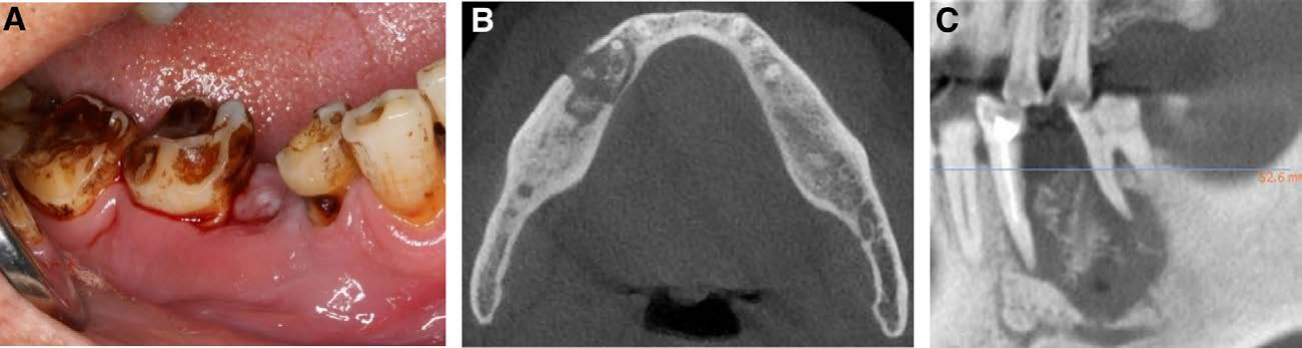
(A) Gingival swelling and pus reappeared. (B) The scope of osteonecrosis of the jaw was significantly enlarged. (C) Bone destruction affected adjacent teeth.

**Figure 5.**
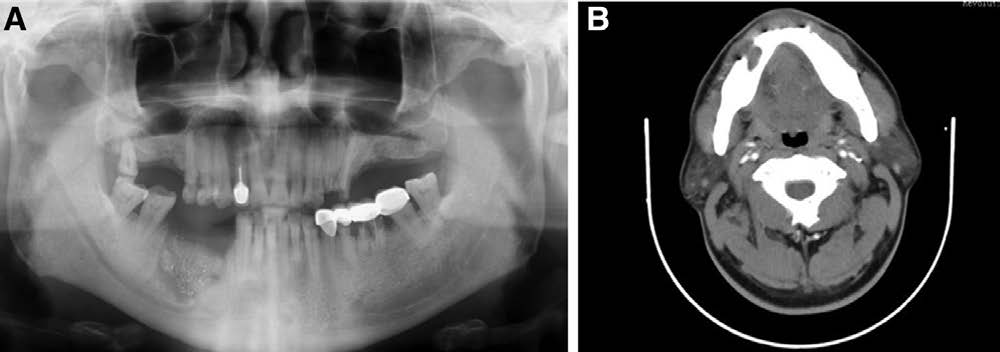
(A) Radiographs after mandibular osteotomy. (B) 3 months after surgery, the cutting boundary was clear.

**Figure 6.**
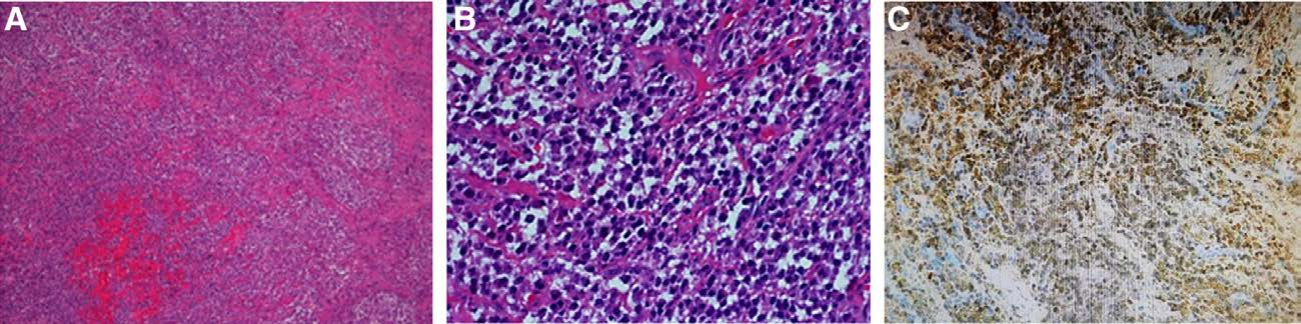
(A and B) Histopathologic section showing massive plasma cell infiltration with hyperchromatic round to ovoid nuclei. (C) Immunohistochemical examination showed CD38(+) and CD 138(+), and light chain staining suggested κ >> λ.

The patient was then lost to follow-up for 10 months, and revisited on November 2021 when gingival swelling and pus reappeared (Fig. 4A). Dental CBCT examination showed that the area affected by osteonecrosis in the right mandible had expanded to the adjacent teeth, causing the loosening of teeth #44 and #46 (Fig. 4B and C). On November 30, 2021, a right mandibular osteotomy was performed, and #44 and #46 was extracted. Radiographs after mandibular osteotomy showed a clear osteotomy margin (Fig. 5A). Postoperative pathological analysis revealed high infiltration of plasma cells in the specimen (Fig. 6A and B). Three months after surgery, the edges of bone defects in the surgical site kept neat (Fig. 5B). Immunological staining confirmed the presence of CD38(+) and CD138(+) plasma cells, and light chain staining suggested κ >> λ (Fig. 6C), indicating that the destruction of the mandible was due to multiple myeloma. Meanwhile, the first pathological report was rechecked by the pathologist, and the recheck results were consistent with the previous pathological results with no abnormal plasma cell infiltration. Three months after the operation, via telephone follow-up, the patient reported that the symptoms of gingival swelling, pain and discharge disappeared after surgery. One year later, through telephone return visit, the patient reported that he was in a stable condition with no signs of recurrence of gum swelling and pain.

## 4. Discussion

Bisphosphonates is a powerful bone absorption inhibitor, which can reduce the risk of MM-related bone fracture and relieve pain, but long-term or high doses of Bisphosphonates treatment increase the risks of MRONJ. Clinical manifestations of MRONJ include pain, exposed necrotic bone or fistula probing into the bone, inflammation of the soft tissue, osteonecrosis, purulent drainage, abscesses, pathological fractures, extraoral fistulas, and oral sinus/oronasal communication.^[12]^ Dental trauma and periodontal disease are the major factors contributing to MRONJ.

MM may occur in the jaw as a solitary lesion or a part of MM especially on the late stage of the disease. The main symptoms are pain, swelling, loose teeth, paresthesia, soft tissue mass, bleeding or pathological fracture.^[13]^ The clinical manifestations and radiology of MM in jaw are non-distinct and variable, which is a challenge to its diagnosis, for oral presentation of MM have similar clinical signs and radiology with MRONJ. The gold standard for the diagnosis of MM is pathological analysis of biopsies.

Several scholars have reported cases of MM misdiagnosed as MRONJ.^[7–11]^ This was partly because biopsies were not usually done for jaws affected by bisphosphonate, In this case, no plasma cell infiltration was found in early specimens of necrotic bone and granulation. but localization of MM in the jaw specimen was observed 1 year later, indicating simultaneously occurring of MRONJ and MM. Ogawa and Katkar et al reported cases of MM in bisphosphonate-affected jaws.^[12,14]^ However, unlike in this case, pathological analysis was not performed at the time of MRONJ diagnosis for the 2 reported cases. A retrospective study reported of the 357 sites of BRONJ related with intravenous bisphosphonate medication subjected to microscopic analysis, 19 (5.3%) sites were diagnosed with 20 cancers in 16 patients in a BRONJ specimen, MM and breast cancer were the 2 main pathologies in the osteonecrosis of the jaws specimens.^[9]^ In this case, MM occurred in jaw might be related to the progression of MM in the late stage. There were reports that the presentation of MM in jaw may be the sign of recurrence or the progression of MM. Invasive treatment such as tooth extraction has been reported to stimulate the development of MM.^[15]^ Meanwhile, The tooth extraction caused MRONJ, and osteonecrosis of the jaw led to further inflammatory spread. The inflammation could be a niche for MM development. To rule out the possibility of inaccuracy in the first pathology report, the pathologist later performed immunohistochemistry on the original sample and verified that there was no abnormal plasma cell infiltration or other tumor cell infiltration. This case suggests that conservative treatment for the stage II MRONJ maybe not effective, so it is necessary to balance the advantages and disadvantages of surgical treatment to avoid long-term inflammation leading to tumor invasion. Meanwhile, patients with MM who develop osteonecrosis after taking bisphosphonates should be screened for MM for the treatment methods of MM in jaw and MRONJ are different. As for MM, Treatments involves chemotherapy, radiotherapy or stem cell transplantation, while patients with MRONJ should be treated with antibiotics and drugs for symptomatic relief. If the 2 diseases are not diagnosed early, it would delay treatment. However, histological examination is not recommended in the diagnosis of MRONJ, except in the case of suspected malignant lesions. In this case, the biopsy was obtained during superficial sequestrum debridement on the patient, not only for purpose of diagnosis. Since the symptoms of osteonecrosis caused by MM are similar to those caused by MRONJ, as well as the presence of gingival fistula and pus in patients diagnosed with MRONJ confused with periodontal abscess. Therefore, detailed clinical analysis supplemented by CBCT, magnetic resonance imaging or fluorodeoxyglucose positron emission tomography/computed tomographic scans even pathological analysis are required to avoid misdiagnosis and missed diagnosis. The limitation of the case was that after the patient was transferred to other hospital, there was no return visit, lacking intraoral photos after surgery.

## 5. Conclusion

These findings suggested that MRONJ and MM can occur simultaneously in jaw. Local inflammation induced by MRONJ could be a niche for MM with bone metastases. Meanwhile, patients presenting with symptoms of MRONJ should be screened for concurrent or disease relapse of multiple myeloma to prevent misdiagnosis or inadequate management.

## Author contributions

**Data curation:** Xiao-Hong Wu.

**Funding acquisition:** Xiao-Hong Wu, Shi-Wei Chen.

**Investigation:** Xiao-Hong Wu.

**Supervision:** Shi-Wei Chen.

**Writing – original draft:** Xiao-Hong Wu.

**Writing – review & editing:** Shi-Wei Chen.
